# Age-Related Microbiota Signatures in Inflammatory Diseases: Comparative Insights into Paediatric and Adult Crohn’s Disease, Ulcerative Colitis, and Spondyloarthritis

**DOI:** 10.31138/mjr.290825.mid

**Published:** 2026-03-01

**Authors:** Giancarlo Gimignani, Alessandro Borello, Cristina Panetta, Linda Lucchetti4, Roberta Caorsi, Marco Gattorno, Alessandro Conforti

**Affiliations:** 1 UOC Medicina Interna, Civitavecchia, Italy;; 2 Gastrointestinal Endoscopy Unit, ASL Roma 4, Civitavecchia, Italy;; 3 ASL Roma D, Lazio, Rome, Italy;; 5 Paediatrics and Rheumatology Clinic, Istituto Giannina Gaslini, Genoa, Liguria, Italy;; 6 Unit of Rheumatology and Autoinflammatory Diseases, Istituto Giannina Gaslini, Genoa, Liguria, Italy;; 7 ASL Roma 4, Civitavecchia, Italy

**Keywords:** Crohn disease, dysbiosis, gastrointestinal microbiome, inflammatory bowel diseases, microbiota, spondylarthropathies

## Abstract

**Objectives:**

The study investigated the relationship among the human microbiota in the development and progression of inflammatory bowel diseases (IBD), specifically Crohn’s Disease (CD) and Ulcerative Colitis (UC), as well as Spondyloarthritis (SpA), comparing paediatric and adult populations.

**Methods:**

The research elaborated the distinct characteristics and impacts of CD, UC, and SpA across age groups. It further explored the developmental stages of the paediatric microbiota, identifying factors like delivery method, feeding, and antibiotics as critical influencers. It examined specific dysbiosis patterns in paediatric IBD and SpA associated to disease activity. Subsequently, it addressed the adult microbiota’s stability and variations due to diet, lifestyle, and medications, detailing microbial alterations in adult CD, UC, and SpA.

**Results:**

A comparative analysis underscores age-related differences in microbiota composition, clinical manifestations, and treatment responses, indicating greater yet weaker microbial populations in adults. In paediatric patients, there was a marked decrease in *Faecalibacterium prausnitzii* and other bacteria responsible for producing short-chain fatty acids. In contrast, adults tended to show a more persistent form of dysbiosis and lower microbiome resilience. These disparities in microbial and metabolic phenotypes were strongly associated with the activity of the disease and the response to the treatment, which suggests the potential of microbiota-based biomarkers to create age-specific diagnostic and therapeutic approaches.

**Conclusion:**

This research found that microbiota play a great role in the inflammatory diseases and they can be of great use in the current treatments as well as serve as a biomarker. The new targeted therapies underscored the necessity of patient specific microbiome studies to enhance diagnostics and therapies of these disorders throughout the lifespan.

## INTRODUCTION

The human body is a multifaceted ecosystem, which lives together with a population of microorganisms which is popularly referred to as human microbiota. This is a community of microbiota that consists of 10 to 100 trillion symbiotic microbial cells which are mostly bacteria, which inhabit mostly the gastrointestinal tract. This definition is further expanded when the term human microbiome is used to describe the totality of the genes that these microbial cells contain.^1^ The human gut has a huge reservoir of microbes containing around 100 trillion microorganisms. This large microbial population is approximated to be 50 to 100 times larger than the human genome and physiologically broadening the physiological capabilities of the host.^2^ The gut microbiota is highly essential in the human body as it helps in digestion and metabolism of nutrients, which essentially increases the metabolic capacity of the host. It plays a central role in the formation of immune systems and determines behavior.^3^ The microbiota has dynamic responses to environmental changes and quickly changes its metabolism. It directly affects host energy balance and has an effect on adiposity by increasing energy extraction. It also highlights the fact that it has a prominent regulatory ability within host physiology and thus, it is a vital target of therapeutic interventions. Gut microbiota is a collection of microorganisms of all three life domains: *Archaea, Bacteria*, and *Eukarya*, as well as viruses. These heterogeneous microbes develop complex feeding interactions with each other and with their human host that may be symbiosis or mutualism as well as parasitism. The human gut microbiota is made up of both autochthonous (indigenous) or native microorganisms and allochthonous (transient) microorganisms.^4^ A recent bibliometric analysis revealed a significant upward trend in publications exploring the link between rheumatic diseases and gut microbiota over the past two decades, highlighting the growing scientific interest in this axis.^5^

The purpose of this review is to build concepts, compare paediatric and adult gut-microbiota signatures in Crohn’s disease, ulcerative colitis, and spondyloarthritis, examine how age-related differences shape disease phenotype, progression, and therapeutic response, and identify opportunities for microbiome-informed, personalised interventions across the life-course.

## OVERVIEW OF INFLAMMATORY DISEASES

### Inflammatory Bowel Disease (IBD)

IBD comprises Crohn’s disease (CD) and ulcerative colitis (UC), two chronic, relapsing conditions of the gastrointestinal tract that together account for much of the global inflammatory-disease burden. Crohn’s disease (CD) produces patchy, transmural inflammation that can occur anywhere from the mouth to the anus, although the terminal ileum and proximal colon are most frequently involved.^5^ The disease often presents with diarrhoea, cramping abdominal pain, weight loss and fatigue; progressive inflammation may lead to strictures, fistulas and extra-intestinal manifestations such as uveitis or arthritis.^6,7^ Therapy is phenotype-directed and ranges from steroid-sparing thiopurines to anti-TNF or other biologics, with surgery required in a substantial proportion of patients.^6^ By contrast, Ulcerative colitis (UC) produces continuous, superficial inflammation that starts in the rectum and extends proximally through the colon. Although pan-colitis can occur at any age, UC is characterised by bloody diarrhoea and urgency rather than the transmural complications typical of CD.^8^ First-line therapy is usually 5-aminosalicylate, with escalation to immunomodulators or biologics as needed for mucosal healing.

### Spondyloarthritis (SpA)

Spondyloarthritis (SpA) is an immune-mediated inflammatory disorder affecting the spine, sacroiliac joints, peripheral joints, and entheses. The key symptoms include severe morning stiffness and chronic back pain that improves with activity. The aetiology of SpA is often associated with the HLA-B27 gene, and symptoms appear with eye inflammation, psoriasis, and inflammatory bowel disease (IBD). The frequent correlation between SpA and IBD suggested a combined inflammatory pathway.^9^

**Table 1.** Key gut-microbiota alterations in paediatric inflammatory diseases, associated metabolic shifts and potential therapeutic relevance.

**Disease**	**Diversity pattern (α / β)**	**Principal taxa**	**Principal taxa**	**Metabolite (functional) changes**	Therapeutic relevance*	**Studies**
Paediatric Crohn’s disease	Marked loss of α-diversity; unstable β-diversity	SCFA-producing Firmicutes (e.g. *Faecalibacterium prausnitzii*, *Blautia*, *Ruminococcus*)	*Bacteroides*, Proteobacteria (*Enterobacterales*), *Veillonella* spp.	Decreased faecal SCFAs, decreased secondary bile acids, decreased serum citric acid; diversity strongly influenced by gastrointestinal transit time (FWC^a)	Serum citric-acid cut-off distinguishes active vs. quiescent CD (AUC 0.77); enrichment of SCFA producers after biologics predicts sustained response	Kong et al., (2025)^28^, Mah et al., (2023)^39^
Paediatric ulcerative colitis	Reduced α-diversity; distinct β-diversity vs. CD	Firmicutes (Clostridia/ Lachnospiraceae), *Anaerostipes*, *Blautia*, *Coprococcus*, *Faecalibacterium*, *Roseburia*, *Ruminococcus*, *Bifidobacterium*	Proteobacteria (*Enterobacteriaceae*), *Enterococcus*, *Haemophilus*, *Veillonella* (incl. oral taxa), *Klebsiella pneumoniae*	Increased tauro-conjugated bile acids; altered plasma polyamines; Veillonella parvula can metabolise calcineurin inhibitors	Post-treatment gain in microbial richness (5-ASA, corticosteroids, biologics) associated with mucosal healing; baseline abundance of *F. prausnitzii* predicts infliximab response	Mah et al., (2023)^39^
Paediatric spondyloarthritis	Dysbiosis with loss of Firmicutes diversity	*F. prausnitzii*, *Clostridium leptum* group	*Bacteroides* spp.; *Akkermansia muciniphila* (subset)	Increased intestinal permeability; sub-clinical gut inflammation; antigenic mimicry with Salmonella typhimurium	Early recognition of microbiota shift may guide diet / probiotic strategies and support rationale for IL-17 blockade in JSpA	Hong S et al., (2025)^26^

* Key clinical applications extracted from the cited studies (e.g. biomarker potential, predictors of therapeutic response, or targets for adjuvant interventions).

## IMPACT OF INFLAMMATORY DISEASES ACROSS AGE GROUPS

### Age-related Differences in IBD

Disease onset before 18 years confers a distinct, often more aggressive phenotype. Children with CD more commonly demonstrate extensive colonic involvement, upper-GI disease, and growth delay, whereas paediatric UC is notable for a high prevalence of pancolitis and relative sparing of the rectum. These factors translate into higher hospitalisation and colectomy rates compared with adult-onset IBD, emphasising the need for early diagnosis and intensive, growth-sparing therapy.^8^

### Age-related Differences in SpA

Juvenile SpA (JSpA) typically emerges in late childhood or adolescence, shows a male predominance, and is driven by peripheral arthritis and enthesitis; axial disease may be clinically silent yet detectable on MRI.^10^ In adulthood, the pattern shifts towards classic ankylosing spondylitis, dominated by spinal and sacro-iliac fusion. This peripheral-to-axial transition is thought to reflect interactions between skeletal maturation, hormonal milieu, and cumulative mechanical stress.^10^ Consequently, functional outcomes are often poorer in JSpA than in adult-onset SpA, reinforcing the importance of early HLA-B27 testing, imaging, and timely initiation of biologic therapy. While IBD and SpA are traditionally studied in separate organ systems, their overlapping epidemiology, shared genetic risk (e.g., HLA-B27, IL-23/IL-17 axis genes), and common response to anti-TNF or IL-17 blockade illustrate how the gut-joint axis links mucosal and musculoskeletal inflammation across the lifespan.^6, 9, 10^

## SEARCH STRATEGY

The literature was searched using different sources including PubMed (MEDLINE), EMBASE, Scopus, and Web of Science from inception to 2025. Search terms included combinations of: *“microbiota”, “gut microbiome”, “dysbiosis”, “inflammatory bowel disease”, “Crohn disease”, “ulcerative colitis”, “spondyloarthritis”, “pediatric”, “adult”*. We also used Boolean operators (AND/ OR), and MeSH terms. Reference lists of identified articles and relevant reviews were screened for additional sources. Only articles in English were included.

### Study Selection

Duplicate records were removed, and two independent reviewers screened articles. Disagreements were resolved by consensus. We did not conduct a formal risk-of-bias scoring given the narrative review format, but we critically appraised conflicting studies and noted methodological limitations.

## MICROBIOTA IN PAEDIATRIC INFLAMMATORY DISEASES

### Development and Influencing Factors of the Paediatric Microbiota

The production of the human gut microbiota is a dynamic and intricate process, with early life representing a critical period for its development and subsequent influence on long-term health. The neonatal period represents a critical window for gut microbial colonisation, during which the immature gastrointestinal tract is rapidly and dynamically populated by diverse microbial communities immediately following birth. During this early developmental phase, the infant gut micro-biome is inherently dynamic and particularly vulnerable to environmental influences.^4^ Perturbations experienced within this period can induce either transient or long-lasting alterations in microbial composition and function, with potential implications for immune development, metabolic health, and future susceptibility to disease.^11^

Several critical factors significantly influence the establishment and subsequent composition of the early-life gut microbiota. The mode of delivery is one of the most significant determinants. The infants born vaginally are typically colonised by maternal vaginal and faecal microbes, which can further facilitate early exposure to beneficial commensals. Comparatively, infants delivered via caesarean section are at significant risk of acquiring microbes from the maternal skin and the surrounding hospital environment. It can lead to delayed or otherwise altered microbial colonisation patterns.^12^ Feeding practices further profoundly shape microbial development throughout infancy. Breast milk is prevalently high in concentration with human milk oligosaccharides (HMOs), immunoglobulins, and live beneficial bacteria, which selectively promote the growth of *Bifidobacteria*.^13^ Bifidobacteria is a genus strongly associated with healthy immune and metabolic development. Conversely, formula-fed infants generally develop a more diverse microbiota, which is often associated with a reduced abundance of *Bifidobacteria*.^14^ It may negatively impact the maturation of both the gut and the immune system. Furthermore, antibiotic exposure during infancy, particularly within the neonatal period, can significantly cause abnormalities in microbial diversity and promote the emergence of antibiotic-resistant strains. These strains have been consistently linked to an increased risk of inflammatory and metabolic disorders.^15^

The maternal factors include diet, microbiota composition, and antibiotic use during pregnancy. It also demonstrated a considerable effect on the colonisation trend of neonates. Furthermore, other environmental exposures, including the presence of domestic pets or siblings that may result in the total number of microbes in the infant gut. These infantile determinants are profound and persistent to the structure and activity of the gut microbiota. It further potentially shapes an individual’s lifelong immune trajectory and their ultimate risk of developing chronic inflammatory conditions.^16^

**Table 2.** Comparative summary of paediatrics and adults in inflammatory diseases.

**Category**	**Paediatric Characteristics**	**Adult Characteristics**	**Overlapping Features / Key Insights**	**Studies**
**Microbiota Development/Stability**	Rapid colonisation post-birth; high plasticity influenced by delivery mode, feeding practices, antibiotic exposure; microbiota matures significantly within first 3 years. Adolescent microbiota uniquely enriched in *Bifidobacterium* and *Clostridium* species.	Established, resilient microbiome with substantial individual stability; resistant to transient perturbations, yet vulnerable to sustained unhealthy shifts ("unhealthy stable states").	Early microbiota establishment critically influences lifelong health; developmental stages shape microbiota-disease interactions.	Davis et al., (2022)^14^, Martínez et al., (2023)^16^, Thriene and Michels (2023)^35^
**Crohn’s Disease: Disease Extent/Severity**	Aggressive phenotype with extensive colonic and upper GI tract involvement; high prevalence of growth impairment and developmental delays.	Typically localised inflammation (terminal ileum predominance); chronic inflammation frequently leads to complications requiring surgical intervention.	Phenotype significantly varies with age of onset; disease severity often correlates inversely with age at diagnosis.	Cockburn et al., (2023)^6^, Day et al., (2012)^8^, Freeman (2014)^42^
**Crohn’s Disease: Complications**	Higher hospitalisation rates; unique paediatric complications (growth failure, delayed puberty); fewer fistulising complications.	Frequent development of strictures, fistulas, bowel obstructions, nutritional deficiencies, malabsorption syndromes, and anal fissures.	Disease complications heavily influenced by age at onset; early aggressive intervention may mitigate severe outcomes.	Cockburn et al., (2023)^6^, Cavalcante et al., (2025)^43^
**Crohn’s Disease: Microbiota Alterations**	Decreased diversity; reduced SCFA-producing Firmicutes (*Faecalibacterium prausnitzii*), increased Proteobacteria (*Enterobacterales*, *Veillonella*), influenced significantly by gastrointestinal transit time.	Consistent dysbiosis with reduced biodiversity; decreased beneficial taxa (*F. prausnitzii, Roseburia hominis*), increased *Enterobacteriaceae*, and fungal dysbiosis (Candida albicans).	Dynamic dysbiosis reflects ongoing inflammation; microbial patterns influenced by gut motility and dietary factors; common reduction in beneficial SCFA-producing bacteria.	Kong et al., (2025)^28^, Fusco et al., (2023)^32^, Sanchis-Artero et al., (2020)^44^
**Ulcerative Colitis: Disease Extent/Severity**	Extensive inflammation with pancolitis predominance (>80%); relative rectal sparing; associated paediatric-specific complications (growth retardation, delayed puberty).	Usually distal and rectum-centric inflammation; increased colorectal cancer risk and severe systemic complications in longstanding disease.	Phenotypic variability strongly age-dependent; distinct paediatric and adult clinical presentations.	Day et al., (2012)^8^, Van et al., (2008)^45^
**Ulcerative Colitis: Complications**	Elevated early risk of hospitalisation and colectomy; notable for paediatric-specific complications affecting growth and puberty.	Increased risk of severe dehydration, colon perforation, osteoporosis, and malignancy (colorectal cancer) with prolonged disease.	Complication profiles shift significantly with age; paediatric-onset demands intensive early therapeutic interventions.	Cabrera and Sato (2018)^46^
**Ulcerative Colitis: Microbiota Alterations**	Significant alpha-diversity loss; depletion of beneficial Firmicutes (*Clostridia*, *Lachnospiraceae*); increased Proteobacteria (*Enterobacteriaceae*), *Enterococcus*, *Veillonella* spp., *Haemophilus*.	Reduced microbial diversity; complex alterations in Firmicutes (variable findings), consistent reduction in anti-inflammatory *F. prausnitzii*, rise in *Enterococcus faecium*.	Common depletion of protective taxa (*F. prausnitzii*); oral-gut microbiota linkage; discrepancies in phylum-level changes reflect methodological variability.	Zhuang et al., (2021)^20^, Alam et al., (2020)^34^, Zhu et al., (2022)^47^
**Spondyloarthritis: Clinical Phenotype**	Predominantly peripheral arthritis and enthesitis; significant hip involvement; often silent axial inflammation detectable via imaging; associated poorer outcomes.	Primarily axial arthritis (spine and sacroiliac joints); chronic inflammatory back pain and progressive ankylosis typical.	Phenotypic progression from peripheral-dominant juvenile form to axial-predominant adult form; indicates developmental modulation of disease expression.	Srinivasalu et al., (2021)^10^, Burgos-Vargas (2012)^48^
**Spondyloarthritis: Microbiota Alterations**	Reduced Firmicutes diversity (*F. prausnitzii, C. leptum* group ); increased Bacteroides spp. and mucin-degrading *Akkermansia muciniphila* subsets; associated subclinical gut inflammation.	Consistent loss of microbiome diversity; decreased *Clostridiales, Lachnospiraceae, F. prausnitzii*; increased *Ruminococcus gnavus* and *Collinsella* species.	Gut-joint axis involvement with shared microbial deficiencies across age groups; consistent dysbiosis linked to disease activity; strong evidence for microbial influence in pathogenesis.	Gill et al., (2018)^23^, Hong et al., (2025)^26^, Cardoneanu et al., (2021)^49^
**Therapeutic Responsiveness**	Enhanced therapeutic responsiveness, notably to diet-based (Exclusive Enteral Nutrition) and microbiota-targeted treatments (FMT); paediatric microbiota shows greater adaptability and plasticity.	Therapeutic responses highly variable; often complicated by chronic, entrenched dysbiosis and cumulative disease complications.	Age-specific windows for microbiota-targeted therapies; early intervention significantly impacts long-term disease trajectory positively.	Mah et al., (2023)^39^, Stiemsma et al., (2018)^37^, Yadav and Chauhan (2022)^50^

### Microbiota in Paediatric Crohn’s Disease

Gut dysbiosis, characterised by disruptions in the composition and function of microbial communities, is strongly implicated in the pathogenesis of paediatric Crohn’s Disease (CD). Studies consistently reported that a decrease in overall microbial diversity and a heightened instability of the gut microbiota in children with CD compared to healthy controls.^17^

Active disease in paediatric Crohn’s disease (CD) is characterised by a marked increase in pro-inflammatory pathobionts, including *Veillonella* and members of the *Enterobacterales* order. It is accompanied by a concurrent decline in beneficial fibre-fermenting commensals. The relative modification in microbial composition determined in relation to disease activity underscores that dysbiosis is not a static phenomenon but rather a responsive marker of ongoing intestinal inflammation. These findings indicate a strong likelihood of the potential utility of the gut microbiome as a non-invasive biomarker for monitoring disease progression and therapeutic response. This also offers a promising adjunct or alternative to conventional diagnostic modalities for inflammatory diseases like Crohn’s disease.^18^ The relationship between the gut microbiome and disease activity in paediatric CD is complex and influenced by the disease activity measure used. Strong correlations are observed with the weighted paediatric Crohn’s disease activity index (wPCDAI) than more objective measures. It is important to note, gastrointestinal transit time, which is reflected in faecal water content, has a strong impact on microbiome signatures in particular with active disease, especially when used with wPCDAI. This implies that alterations of gut motility could be a secondary cause of microbial changes.^19^

### Microbiota in Paediatric Ulcerative Colitis

Paediatric Ulcerative Colitis (UC) can be defined by a complicated combination of immune dysregulation, imbalance of microbiota and dysfunction of the intestinal barrier. The research studies examining the gut microbiota in childhood UC all resource reports a high level of alpha-diversity indices, meaning that the richness and total diversity of the microbial community has reduced relative to healthy controls. In addition to internal diversity, it is also found that differences in beta-diversity (that measures the overall community structure of samples) exist between paediatric UC patients and healthy individuals, indicating that paediatric UC patients may have a different microbial composition than healthy individuals.^14, 19^

Regardless of methodological differences, bacterial changes in paediatric UC are more likely to include a reduction in Firmicutes (*Clostridia* and *Lachnospiraceae*) and an expansion in Proteobacteria (*Enterobacteriaceae*). Certain genera, such as *Anaerostipes, Blautia, Faecalibacterium* and *Roseburia* are usually decreased and *Enterococcus, Haemophilus* and *Veillonella* are enriched. Moderate to severe UC children exhibit an increased oral cavity microflora (e.g., *Veillonella, Haemophilus parainfluenzae, Klebsiella pneumoniae*) and reduced *Bifidobacteria*.^20^

Metabolically, severe childhood UC is linked to higher tauro-conjugated bile acid and lower plasma tryptophan metabolites and polyamines, which indicate that there is a lack of immune control. It is worth noting that Veillonella parvula, which increases in severe cases of UC, has the potential to catabolise immunosuppressive medications, which may affect the effectiveness of treatment. The interaction between gut dysbiosis and drug metabolism outlines the complexity of the paediatric UC management process and the need to develop individualised treatment plans.^21^

### Microbiota in Paediatric Spondyloarthritis

There is mounting evidence to suggest that gut microbial dysbiosis is the cause of various inflammatory diseases, one of them being Spondyloarthritis (SpA). Subclinical gut inflammation triggered by changes in intestinal microbial balance is believed to be the start of immune activation at the entheses and joints in juvenile SpA (jSpA). This gut–joint connection underscores the importance of early microbial disturbances in shaping systemic inflammatory responses.^22^

Across multiple studies, a consistent finding in patients with juvenile spondyloarthritis (jSpA) is a reduced abundance of *Firmicutes*, a dominant phylum of beneficial gut commensals. Additionally, key anti-inflammatory species such as *Faecalibacterium prausnitzii* and *Clostridium leptum* are significantly decreased in jSpA, which is a pattern that closely parallels observations in adult SpA and inflammatory bowel disease (IBD).^23, 24^

The recurrent depletion of *F. prausnitzii* in both paediatric SpA and IBD represents a compelling cross-disease signature. The major producer of butyrate, a short-chain fatty acid with well-documented immunoregulatory effects, *F. prausnitzii* plays a vital role in maintaining intestinal immune homeostasis, in part by supporting regulatory T cell function. Its lesser abundance can be an indication of a common microbial susceptibility, which leads to systemic inflammation, whether in the form of gut pathology or joint involvement or both. These results also highlight why microbial restoration and specifically the restoration of butyrate-producing taxa such as *F. prausnitzii* is a viable area of therapeutic activity in a range of inflammatory disorders.^25^

Besides, the microbiota of jSpA patients have shown that they can be organised in various microbial clusters. One of the clusters is mainly represented by the representatives of the Bacteroides genus, another one is represented by *Akkermansia muciniphila*. It is also interesting to note that the abundance of *A. muciniphila* is higher, because it is capable of degrading intestinal mucins. It is also capable of undermining the gut barrier and causing a rise in permeability, which is seen in both jSpA and adult SpA.^26^

Studies were highly indicating the existence of a gut-joint axis during paediatric SpA, in which gut dysbiosis and impaired intestinal barrier are precursors to systemic inflammation. Juvenile SpA is associated with an increased intestinal permeability relative to other types of JIA as well as healthy controls, which may permit the participation of microbial components in systemic immune responses involving joints. Even without symptoms of gastrointestinal problems, subclinical gut lesions in these children are demonstrated by the use of colonoscopy, faecal calprotectin, and MRI. This association is further supported by a cellular immune response to *Salmonella typhimurium* and a lowered anti-inflammatory *Faecalibacterium prausnitzii*. The results suggest gut microbial or dietary intervention as a preventive or curative intervention in paediatric SpA to re-establish gut barrier integrity and re-organise the microbial ecosystem to diminish systemic inflammation.^27^

## MICROBIOTA IN ADULT INFLAMMATORY DISEASES

### Stability and Variations of the Adult Microbiota

Unlike the microbiota of early childhood which is highly dynamic and developing, the human fecal microbiome of healthy adult life is characterised by an astonishing level of stability and consistency over time, and can last years and even decades. It has been demonstrated that within-person taxonomic and functional variation in the adult microbiome is consistently lower than the variation observed between different individuals over time. It implies that, the microbiome of an individual is likely to be more like its own previous states than that of an individual, meaning that each person has a distinct microbial signature.^27^

Adult microbiome stability varies; functional potential (DNA) is most stable, followed by gene expression (RNA), and then species composition. While resilient to transient changes like antibiotics, it can become stuck in an unhealthy state, unlike the highly plastic infant microbiome where early disruptions have lasting effects. Healthy adult microbiomes are generally more stable due to functional redundancy, where multiple species perform similar roles, providing a buffer against species loss. This underscores the importance of early microbiome establishment in infancy, as the window for reprogramming narrows in adulthood.^28, 29^

### Factors Influencing the Adult Microbiota

Diet significantly and rapidly modulates the adult gut microbiota. Protein and animal fat-rich diets correlate with *Bacteroides*-driven profiles, while carbohydrate-rich diets link to *Prevotella*. Plant-based diets, rich in MACs, increase microbial diversity and beneficial butyrate-producing bacteria like *Roseburia* and *Faecalibacterium*. Low-MAC Western diets reduce diversity. Medications, particularly antibiotics, cause substantial and lasting changes. Other drugs and xenobiotics, like artificial sweeteners and emulsifiers, can also modify the microbiota. Lifestyle factors such as exercise can increase diversity and beneficial bacteria. Stress can reduce beneficial bacteria and increase gut permeability. Geographic location and ethnicity also influence microbiota composition due to varying diets and environments. These factors highlight the significant individual control over adult gut health, suggesting personalised lifestyle interventions for managing inflammatory diseases.^30^

### Microbiota in Adult Crohn’s Disease

Dysbiosis, which is characterised by significant alterations in the gut microbiome, is a consistent finding in adult Crohn’s Disease (CD) patients when compared to healthy controls. The patients with CD typically present with reduced biodiversity within their gut microbial communities. There are some studies that report up to 25% fewer taxa than healthy individuals.^31^

The common and recurrent alterations include a notable decrease in the abundance of bacteria belonging to the phylum *Bacillota* (formerly *Firmicutes*), specifically beneficial species such as *Faecalibacterium prausnitzii* and *Roseburia hominis*. *F. prausnitzii* is a particularly vital beneficial species due to its known anti-inflammatory properties in the gut. Alternatively, there is an increase in potentially pathogenic taxa, such as *Enterobacteriaceae*, that is frequently observed in CD patients. Microbiota profiles can also vary depending on the specific IBD subtype and disease phenotype, with changes detectable during both active and quiescent disease states.^32^

The dietary patterns play a crucial role in shaping dysbiosis of the human gastrointestinal tract. High-fat diets (HFDs) can disrupt gut homeostasis by increasing the *Firmicutes/Bacteroidetes* ratio, which is often associated with adverse health outcomes. HFDs also lead to a reduction in the production of beneficial short-chain fatty acids (SCFAs) and an increase in endotoxemia, which can ultimately contribute to intestinal dysbiosis and a compromised epithelial barrier function. The presence of fungal dysbiosis is also observed, aside from bacterial dysbiosis with an increased abundance of fungal colonies. *Candida albicans*, has been linked to a lack of response to advanced therapies in CD, highlighting the importance of the mycobiome in disease pathogenesis and treatment outcomes.^33^

### Microbiota in Adult Ulcerative Colitis

Ulcerative colitis (UC) is a chronic inflammatory bowel disease marked by a complex interplay of immune dysregulation, gut barrier dysfunction, and significant microbial imbalance. A consistent hallmark in adult UC patients is a reduction in overall bacterial diversity, as evidenced by decreased species richness and Shannon diversity indices compared to healthy controls.^34^ However, the direction and magnitude of phylum-level shifts remain debated, with studies frequently reporting conflicting results. For example, while Alam et al. observed a pronounced increase in the phylum Firmicutes in UC patients relative to controls, this finding diverges from several global studies where Firmicutes abundance is often unchanged or even decreased in IBD cohorts. Such discrepancies likely reflect variations in study populations, sequencing methods, and sample sizes, highlighting the need for cautious interpretation and the recognition of inter-study variability.^34^ In the study by Alam et al., Firmicutes and Actinobacteria were notably elevated in UC patients, while Proteobacteria and Bacteroidetes showed a relative decrease compared to controls. Within Firmicutes, increases in the classes *Clostridia*, *Negativicutes*, and *Bacilli*, and families such as *Ruminococcaceae* and *Lachnospiraceae*, were detected. Conversely, some Firmicutes families, including *Acidaminococcaceae* and *Lactobacillaceae*, were reduced, illustrating the complexity of these taxonomic shifts. Notably, beneficial taxa such as *Faecalibacterium prausnitzii* are consistently reduced in UC, which may impact anti-inflammatory short-chain fatty acid production. There is also an increase in potentially pathogenic species, such as *Enterococcus faecium*, and a decrease in certain Bacteroidetes families (*Rikenellaceae* and *Tannerellaceae*). Importantly, the altered microbiota in UC is not only less diverse but also less functionally integrated, with many taxa that increase in abundance being poorly connected to the healthy gut microbial network. This disrupted co-abundance likely contributes to impaired resilience and further intestinal inflammation, underscoring the importance of both diversity and microbial network stability in UC pathogenesis.^34^

### Microbiota in Adult Spondyloarthritis

Gut microbiome has a significant role to play in SpA, and variation in diversity and individual bacteria are associated with disease activity. HLA-B27 gene has been found to alter the gut microbiota, and it suggests that there is a genetic-microbial axis in the pathogenesis of SpA, and that specific microorganisms may be targeted in personalised therapy. Weakened gut barrier permits the entry of inflammatory molecules to circulation worsening SpA. Reduced *Clostridiales* and microbial diversity and increased *Ruminococcus gnavus* are always studied and associated with disease activity respectively. The anti-inflammatory bacteria, *Faecali-bacterium prausnitzii*, are often decreased. Causal relationships between particular taxa such as *Actinobacteria* id.419 (raised AS risk) and *Rikenellaceae* id.967 (reduced AS/PsA risk) were found using Mendelian randomisation. The microbial profiles are associated with the disease activity scores, which suggests their use as severity and progression markers.^35^

## DISCUSSION

### Age-Related Differences in Microbiota Composition and Function

There is dynamic development of the human gut micro-biota, which starts at birth, and the initial colonisation depends on the delivery mode, type of feeding, exposure to antibiotics and environmental contacts. Micro-biota during the period of infancy and early childhood are highly plastic and volatile, stabilising at the age of three. The adolescent microbiota is distinct in composition, characterised by an abundance of genera that are highly fermentative (*Bifidobacterium* and *Clostridium*), which differentiate the paediatric microbiota not only in infancy but also in adulthood.^36^ Physiologically, the adolescent microbiota is higher in fermentative activity, resulting in elevated concentrations of short-chain fatty acids (SCFA), which are fundamental to epithelial wellness and immunosuppression. Conversely, adult microbiota exhibit greater compositional stability and less resistance to chronic stressors like chronic inflammation or poor diet.^37^ The interaction of sex hormones and the gut microbiome has been involved in systemic autoimmunity that refines the idea that microbial community structure can be modulated by hormonal impacts across multiple times in life.^38^ These developmental patterns demonstrate the significance of life-course approach in the interpretation of host-microbe interactions in the pathogenesis of inflammatory diseases.

### Disease Progression Across Age Groups

The age at which disease occurs is an important factor that will determine clinical progression and complications of inflammatory diseases. Paediatric IBD, especially, Crohn’s Disease and UC, tends to have a wider and more aggressive presentation as compared to adult-onset disease. IBD in children is often characterised by pancolitis, involvement of the upper GIT tract, and impaired growth, and should receive early and intensive treatment.^6,8^ Genetic predispositions, including NOD2 or IL23R mutations, have more significant effects in early childhood, and accumulating environmental influences and epigenetic effects with age. The aggressive phenotype does not necessarily result in worse mucosal healing and therapeutic response by paediatric patients, especially in response to exclusive enteral nutrition (EEN) and first-line biologics. By comparison, adult-onset IBD has a higher likelihood of being cumulative structural damage, fistulising disease, and colorectal malignancy. Peripheral arthritis, hip involvement, and enthesitis dominate JSpA in Spondyloarthritis, whereas axial inflammation (that is detectable by MRI) is common in adult-onset SpA and tends to be associated with poorer long-term prognosis.^10^ Adolescence is associated with hormonal shifts and biomechanical alterations that can contribute to the disease phenotype, and therefore, age-specific screening and treatments are necessary.

### Therapeutic Implications Arising from Age-Related Microbiota Differences

The plasticity in the development of the microbiota presents a therapeutic opportunity in paediatric patients. EEN, probiotics, as well as faecal microbiota transplantation (FMT), seem more useful in young patients, where they usually have a significant effect on clinical outcomes and mucosal healing.^37,39^ In rheumatic disease, immunonutritional approaches addressing microbiota and micronutrient balance have been investigated with the same remission rate and few side effects as corticosteroids, and early microbial interventions can also minimise potential complications such as stricturing or fistulisation.^40^ In paediatric CD, EEN can be used with comparatively high remission rates and fewer adverse effects than corticosteroids, and early microbial interventions may help decrease the risk of complications, such as stricturing or fistulisation. In adults, by contrary, dysbiosis is usually entrenched, the barrier is malfunctioning, and comorbidities can lead to a poor ability to respond to microbiota-targeted therapy. The relative stability in the adult microbiome in response to change and cumulative inflammation often requires increased therapeutic regimens. New diagnostic microbiome-derived tools, e.g., *F. prausnitzii* count, or enteric metabolomic profiling, can enhance the selection of treatment by age. To confirm the utility of these instruments, age-stratified clinical trials are crucial in order to improve therapeutic algorithms.

The literature on the gut-joint axis, the significance of immunonutrition, and the effect of microbiome in rheumatic diseases has been mainly covered in earlier studies of the Mediterranean Journal of Rheumatology (MJR). Yet, such studies have been conducted on individual disease categories or on the adult population. It is a comparative age-stratified synthesis of microbiota composition and activity in Crohn Disease, Ulcerative Colitis, and Spondyloarthritis, with particular focus on the distinctions between childhood-onset and adult-onset disease. It combines the evidence of gastroenterology and rheumatology, establishing connections between clinical and therapeutic outcomes and the development of microbiomes.

Faecal microbiota transplantation (FMT) has become one of the prospective microbiome-based interventions to restore intestinal microbial homeostasis by transferring a donor faecal material of healthy subjects to those with the condition. Although FMT is proven to be an effective therapy against Clostridioides difficile infection, there is still research on its use to treat inflammatory bowel diseases (IBD). Varied results of clinical researches in ulcerative colitis have indicated varied remission rates, which in many cases depend on the diversity of donor microbes, preparation procedures, and disease activity during treatment. In Crohn, the data is not as consistent with some studies reporting the short-term inflammation markers improvements but no long-term remission.

In addition to IBD, there is preliminary evidence that FMT can alter systemic immune responses in autoimmune inflammatory conditions including Spondyloarthritis, multiple sclerosis and type 1 Diabetes. Facing the potential, FMT has a number of challenges such as variable donors, risk of pathogen transmission, lack of standardised preparation and administration procedures, and are unclear regarding the optimal frequency or dosage. Ethical and regulatory issues are also still in existence, especially when it comes to long-term safety and usage in paediatric populations. The solution to these shortcomings is in the development of defined microbial consortia (so-called next-generation probiotics) and synthetic stool substitutes, which will be more controlled and reproducible, providing a greater range of therapeutic options in clinical practice.

## IMPACT OF BIOLOGICAL THERAPIES ON GUT MICROBIOTA

The biological therapies, especially anti-TNF agents, anti-IL-12/23, and anti-integrin monoclonal antibodies are not only changing the way inflammatory bowel disease (IBD) and Spondyloarthritis (SpA) are treated but are also more and more acknowledged to have a secondary impact on the gut microbiota composition. Anti-TNF therapy with infliximab, adalimumab, and other treatment has been linked to be partially restoring microbial diversity and expanding of beneficial taxa, including *Faecalibacterium prausnitzii*, *Roseburia*, and *Blautia*. These modifications are frequently related to the enhanced healing of the mucosa and clinical remission, which indicates a two-way interrelation between immune regulation and microbial homeostasis.

## DIETARY INTERVENTIONS AND MICROBIOTA MODULATION

Diet has a key contribution in the composition of gut microbiota and metabolic output, which is the foundation of prevention and intervention of inflammatory diseases. Of eating patterns, the Mediterranean diet (high fruits, vegetables, whole grains, legumes, nuts, olive oil, and fish) is associated with enhanced microbial diversity, enhancement of short-chain fatty acid-generating taxa (*Faecalibacterium prausnitzii* and *Roseburia*) and depletion of pro-inflammatory bacteria. Compliance with this diet has been linked with decreased disease severity and better quality of life in patients with IBD and rheumatic diseases. On the other hand, low-FODMAP diets (fermentable oligosaccharides, disaccharides, monosaccharides, polyols) are able to reduce functional gastrointestinal symptoms in IBD, at the cost of possibly reducing beneficial microbial populations of fermentables and butyrate synthesis. Although gluten-free diets are necessary in celiac disease, they produce inconsistent results in non-celiac inflammatory diseases; temporarily, they might decrease the microbial diversity and increase or decrease the levels of *Bifidobacterium*.

## FUTURE DIRECTIONS AND RESEARCH GAPS

Regardless of significant progress, there are still a number of gaps in our knowledge about the age-related microbiota in inflammatory diseases. In IBD and SpA longitudinal, birth-cohort studies with standardised sampling guidelines are required to track the progression of microbiota since infancy into adulthood. The study of long-COVID supports the idea that long-term microbiota changes are potentially associated with various disease outcomes, as the limited number of studies conducted to date combine diet, host genetics, metabolomics, and immune profiling across age groups.^41^ Further, the differences in sequencing technologies and analytical pipelines make comparisons of cross-studies more challenging, especially those at the phylum and genus levels. The other area that has not been studied properly is how sex hormones, microbial ecology, and immune tolerance interact in the context of puberty, an important phase of development. Gender-specific interventions can be guided by the understanding of how puberty-related changes contribute to the appearance of diseases. The current research is not as confirmative as a systematic review. The possible biases are selection bias, publication bias, and inter-study heterogeneity. We tried to overcome these through extensive database searching, including of competing evidence, and making clear a report of our search strategy. Lastly, microbiota-based therapies including bacteriophage therapy, SCFA supplementation, and strain-specific probiotics have potential but have not been validated in regards to efficacy and safety in populations of children. To facilitate individual, life-course treatment of inflammatory diseases, future studies should focus on age-adapted interventions and microbial biomarkers.

## CONCLUSION

The microbiota plays a significant role in health, and its lack of balance (dysbiosis) is associated with inflammatory ailments such as Crohn disease, Ulcerative Colitis, and Spondyloarthritis. The knowledge of micro-biota age variations is crucial in treatment; microbial profiles and their influence may be different in children and adults. The field of microbiome profiling in diagnostics has the potential to predict the treatment response and introduce individual therapy to address the healthcare issue, as well as save medical expenditures. New microbiota-directed therapies are heterogeneous. Probiotics and prebiotics strive to develop healthy microbial communities. Faecal Microbiota Transplantation (FMT) demonstrates efficacy in restoring gut homeostasis, especially C. difficile infections and is in trial in IBD and SpA. More tools can be rationally-defined bacterial consortia (Live Biotherapeutic Products), application of a particular bacterial metabolite (microbial substrates) to anti-inflammatory purposes. The more complicated techniques include the editing of microbiota using bacteriophages or more highly engineered bacteria to provide therapeutics. There is also a significant effect of dietary modulation, such as paediatric Crohn’s on exclusive enteral nutrition on the microbiome. The directions of future studies should be to develop individual therapies, determine cause and effect relationships between changes in the microbes and the disease, and determine sex-specific microbial signatures.
